# Proton transfer and conformational changes along the hydrogen bond network in heliorhodopsin

**DOI:** 10.1038/s42003-022-04311-x

**Published:** 2022-12-06

**Authors:** Masaki Tsujimura, Yoshihiro Chiba, Keisuke Saito, Hiroshi Ishikita

**Affiliations:** 1 Department of Advanced Interdisciplinary Studies, The University of Tokyo, 4-6-1 Komaba, Meguro-ku, Tokyo 153-8904 Japan; 2 Department of Applied Chemistry, The University of Tokyo, 7-3-1 Hongo, Bunkyo-ku, Tokyo 113-8654 Japan; 3 Research Center for Advanced Science and Technology, The University of Tokyo, 4-6-1 Komaba, Meguro-ku, Tokyo 153-8904 Japan

**Keywords:** Bioenergetics, Biophysical chemistry

## Abstract

Heliorhodopsin releases a proton from the Schiff base during the L-state to M-state transition but not toward the protein bulk surface. Here we investigate proton transfer and induced structural changes along the H-bond network in heliorhodopsin using a quantum mechanical/molecular mechanical approach and molecular dynamics simulations. Light-induced proton transfer could occur from the Schiff base toward Glu107, reorienting Ser76, followed by subsequent proton transfer toward His80. His80 protonation induces the reorientation of Trp246 on the extracellular surface, originating from the electrostatic interaction that propagates along the transmembrane H-bond network [His80…His23…H_2_O_[H23/Q26]_…Gln26…Trp246] over a distance of 15 Å. Furthermore, it induces structural fluctuation on the intracellular side in the H-bond network [His80…Asn16…Tyr92…Glu230…Arg104…Glu149], opening the inner cavity at the Tyr92 moiety. These may be a basis of how light-induced proton transfer causes conformational changes during the M-state to O-state transition.

## Abstract (web summary)

QM/MM and MD simulations reveal the proton transfer pathway and induced structural changes along the hydrogen bond network in heliorhodopsin 48C12.

## Introduction

Heliorhodopsins, a class of microbial rhodopsins, are widely present in archaea, bacteria, eukaryotes, and algal viruses^1^. The functions of heliorhodopsins remain unclear. Heliorhodopsins may be a photosensor because of its long photocycle (>1 s)^1–3^, or function as a transporter of molecules that are not permeable across the outer membrane of diderms (e.g., amphiphilic compounds), as they are not present in diderms^4,5^.

Heliorhodopsins have an all-*trans* retinal chromophore that is covalently attached to a conserved lysine residue via a Schiff base, with the photoisomerization of the retinal chromophore to the 13-*cis* configuration driving the photocycle^1^. Remarkably, they are embedded in the membrane with an inverted topology, wherein the N- and C-terminals are found on the intracellular and extracellular sides, respectively, unlike other microbial rhodopsins^1^. Therefore, they share little sequence similarity with other microbial rhodopsins. The X-ray structure of bacterial heliorhodopsin 48C12 shows that the Schiff base forms an H-bond with Glu107^6^. Glu107 also forms an H-bond via a cluster of water molecules with an H-bond chain (Tyr92, Asn16, His80, His23, Gln26, and Trp246) that proceeds from the intracellular side toward the extracellular side along the transmembrane helices^6^. Tyr92 forms an H-bond network with Glu230, Arg104, and Glu149 on the intracellular side, whereas Trp246 exists as a terminal site on the extracellular side.

In heliorhodopsin, the deprotonation of the Schiff base occurs during the L-state to M-state transition^1^. However, the absence of light-induced pH changes indicates that heliorhodopsin does not release the proton toward the protein bulk surface^1,7^. This suggests that the proton released from the Schiff base remains at the terminal proton acceptor in the protein interior in the M state. Glu107 is involved in proton transfer during M-state formation. According to the observations in time-resolved resonance Raman spectroscopy^8^ and Fourier transform infrared (FTIR) spectroscopy^9^, the strength of the H-bond between the Schiff base and Glu107 increased prior to Schiff base deprotonation. However, Glu107 is not the terminal proton acceptor in the M state, as the C=O stretching frequency of protonated carboxylate was not observed by FTIR spectroscopy^1^. The M state can form in the E107Q, H80F, H23F^1^, E149Q, and E230Q^6^ mutant heliorhodopsin 48C12. The formation of the M state was also observed in the H23F/H82F mutant heliorhodopsin from *Thermoplasmatales* archaeon SG8-52-1, which corresponds to the H23F/H80F mutant heliorhodopsin 48C12^2^. The photocycle was significantly slowed down by H23F and H80F mutations^1^. His23 and His80 are important for proton transfer^1,10^, although His23, His80, and Glu107 may not necessarily act as a terminal proton acceptor from the Schiff base^6^. Kovalev et al. proposed that a cluster of water molecules near Glu107, His80, and Glu230 might serve as a proton reservoir in the M state^6^. This proposal resembles that of bacteriorhodopsin, where a proton might be delocalized over a cluster of water molecules near Tyr57, Arg82, Tyr83, Glu194, and Glu204^11,12^. According to FTIR studies, significant conformational changes occur in the M-state to O-state transition^1^. The O state did not form in the H80F mutant^1^. These imply that histidine residues play a role in M-state and O-state formation. Here, we investigated proton transfer and induced structural changes along the H-bond network in heliorhodopsin 48C12, using a quantum mechanical/molecular mechanical (QM/MM) approach and molecular dynamics (MD) simulations.

## Results

### Energetics of proton transfer in the H-bond network

**Fig. 1:**
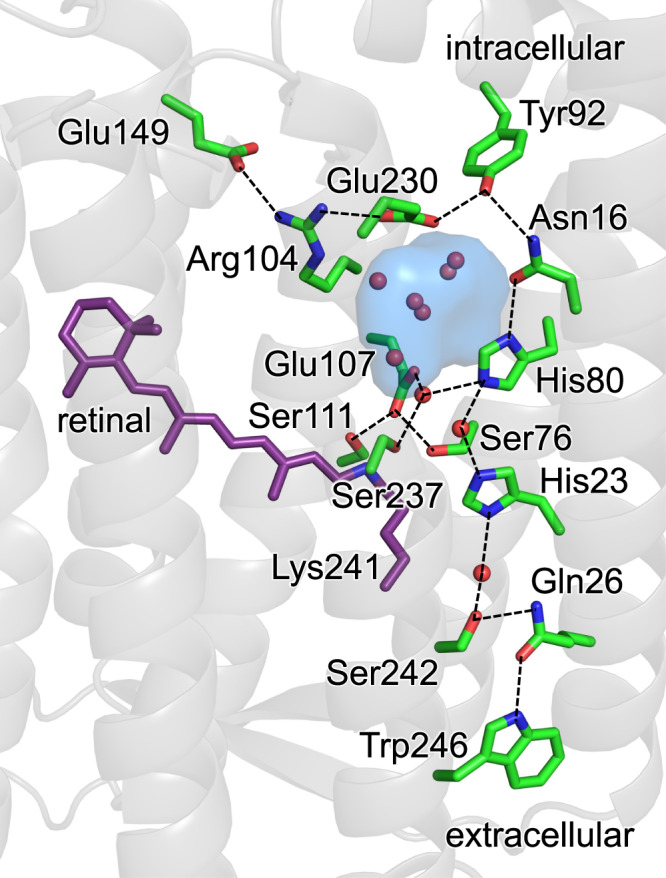
H-bond network that proceeds from the Schiff base via Glu107 in heliorhodopsin 48C12. Dotted lines indicate H-bonds. The cavity filled with a cluster of water molecules is shown as a blue surface.

**Fig. 2:**
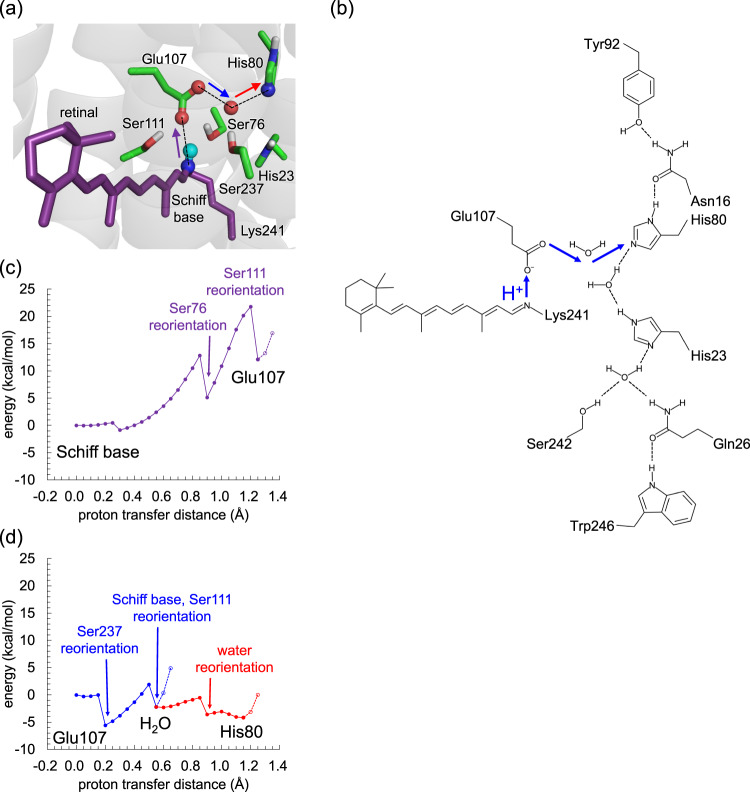
Energetics of proton transfer in the H-bond network. **a**, **b** H-bond network of the Schiff base. **c** Potential energy profile for proton transfer from the Schiff base to Glu107 in the ground-state structure of heliorhodopsin. **d** Potential energy profile for proton transfer from Glu107 to His80. Purple, blue, and red curved lines in (**c**) and (**d**) correspond to the purple, blue, and red arrows in (**a**).

The potential energy profile for proton transfer from the Schiff base to Glu107 indicates that the proton is predominantly localized at the Schiff base moiety in the ground-state structure (Figs. 1, 2a–c): that is, p*K*_a_(Schiff base) > p*K*_a_(Glu107), independent of the protonation states of His23 and His80 (i.e., in all possible histidine protonation conformations) (Supplementary Fig. 1).

First, the energetics of proton transfer are investigated, using a QM/MM approach based on the ground-state conformation with the all-*trans* retinal Schiff base (Fig. 2). To the best of our knowledge, intermediate-state structures have not been reported for heliorhodopsins. In addition, the H-bond between the Schiff base (the proton donor) and Glu107 disappears in the MD-generated structure with the deprotonated 13-*cis* retinal Schiff base, preventing us from investigating the energetics of the initial proton transfer process (Supplementary Fig. 2). Although the energy profile for proton transfer in the ground-state structure may be energetically more uphill than that in the relevant intermediate structure, analysis using the ground-state crystal structure is the best starting point and is partly justified by experimental evidence (e.g., serine conformation, see below).

#### Before proton transfer from the Schiff base to Glu107

Three serine residues, namely, Ser76, Ser111, and Ser237, are highly conserved among heliorhodopsins in the Schiff base moiety (Figs. 1, 2a)^6^. Serine hydroxyl groups can serve as an H-bond donor and acceptor. However, the OH orientation in the original X-ray diffraction structure (at 1.5 Å resolution^6^) is unclear due to the absence of H atoms.

QM/MM calculations indicate that Ser111 donates an H-bond to Glu107, stabilizing deprotonated Glu107 and thereby contributing to p*K*_a_(Schiff base) > p*K*_a_(Glu107) (Fig. 2a). The polar hydroxyl O atom of Ser237 orients toward the Schiff base, stabilizing protonated Schiff base and also contributing to p*K*_a_(Schiff base) > p*K*_a_(Glu107) (Fig. 2a). QM/MM calculations show that Ser111 and Ser237 contribute to the decrease in the absorption wavelength of 5 and 12 nm (Table 1, Supplementary Fig. 3), which suggests that mutations of these serine residues to alanine would increase the absorption wavelength by 5 and 12 nm, respectively. Consistently, mutational studies showed that mutations of Ser111 and Ser237 to alanine increase the absorption wavelengths by 8 and 10 nm, respectively^10^. Furthermore, the absorption wavelength calculated using the conformation wherein Ser111 donates an H-bond to Glu107 and the polar hydroxyl O atom of Ser237 orients toward the Schiff base is 557 nm, which reproduces the experimentally measured absorption wavelength of 551 nm^1^. These results confirm that when the Schiff base is protonated, Ser111 donates an H-bond to Glu107 and the polar hydroxyl O atom of Ser237 orients toward the Schiff base, both contributing to the fixation of the proton at the Schiff base moiety in the ground-state structure.

**Table 1:** Contributions of serine residues to the absorption wavelength of the retinal Schiff base (*λ*) in nm.

Serine	Contribution to *λ*	Conformation
Ser76	−9	pre-PT_SB→E107_^a^
	+4	post-PT_SB→E107_^b^
Ser111	−5	pre-PT_SB→E107_
Ser237	−12	pre-PT_SB→E107_

^a^Ser76 orients toward Glu107 (Fig. 3).

^b^Ser76 orients toward His23 (Fig. 3).

#### During/after proton transfer from the Schiff base to Glu107 (PTSB→E107)

**Fig. 3:**
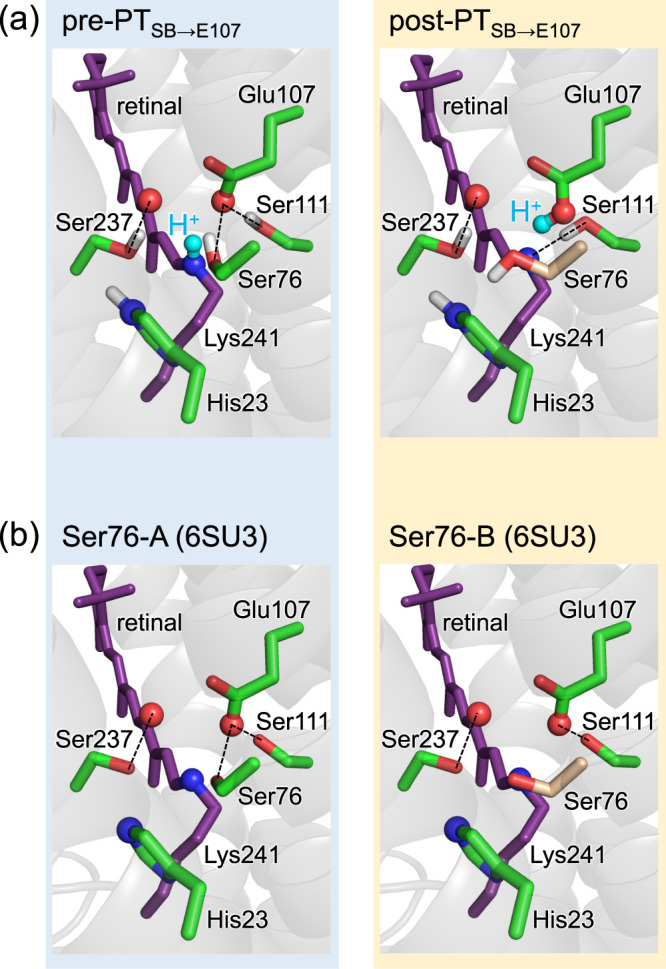
Two distinct Ser76 conformers. **a** Pre-PT (left panel) and post-PT (right panel) conformations observed during proton transfer from the Schiff base to Glu107 in QM/MM calculations. **b** Ser76-A (left panel) and Ser76-B (right panel) conformations identified in the heliorhodopsin crystal structure (PDB code 6SU3)^6^. Dotted lines indicate H-bonds.

QM/MM calculations show that Ser76 donates an H-bond to Glu107 in the ground-state structure (pre-PT_SB→E107_ conformation, Fig. 3a). According to the QM/MM calculations, Ser76 contributes to the decrease in the absorption wavelength of 9 nm (Table 1, Supplementary Fig. 3), which is consistent with the increase in the absorption wavelength of 6 nm observed upon the S76A mutation^10^. Thus, Ser76 donates an H-bond to Glu107 in the ground state together with Ser111.

Although proton transfer occurs during the L-state to M-state transition^1^, conformational changes are observed in response to the movement of the proton from the Schiff base toward the Glu107 moieties in the ground-state structure. As the proton moves to Glu107, Ser76 reorients toward His23 (post-PT_SB→E107_ conformation, Fig. 3a), and the energy significantly decreases (Fig. 2c). (Note: Ser76 cannot form an H-bond with His23 because the hydroxyl group of Ser76 is not in the same plane as that of His23; Fig. 3a). Remarkably, the resulting post-PT_SB→E107_ conformation, wherein Ser76 orients toward His23, is substantially identical to the alternative conformation of Ser76 presented in the crystal structure (designated as “B” in PDB code 6SU3^6^, Fig. 3b). Consistently, the post-PT_SB→E107_ conformation was identified in the structure crystallized at pH = 4.3, where a proton may be shared between Glu107 and acetate^6^.

These results suggest that the reorientation of Ser76 is a prerequisite for proton transfer from the Schiff base to Glu107, and that the alternative Ser76 conformation identified in the ground-state crystal structure^6^ represents the post-PT_SB→E107_ conformation. Notably, Ser76 in heliorhodopsin is substantially conserved as Ser70 in Na^+^-pumping rhodopsin KR2, where Ser70 donates an H-bond to the counterion (Asp116) in the ground-state structure^13,14^ and it reorients during the proton transfer from the Schiff base to Asp116 in the L-state to M-state transition^15,16^.

As the proton reaches the Glu107 moiety, Ser111, which also donates an H-bond to Glu107 in the presence of the protonated Schiff base, reorients and forms an H-bond with the deprotonated Schiff base, facilitating proton fixation at the Glu107 moiety (Fig. 3a), although Ser237 does not reorient during the proton transfer from the Schiff base to Glu107 (Fig. 3a) (see below for further details).

#### Proton transfer from Glu107 to His80 (PT_E107→H80_)

Glu107 serves as a proton acceptor for the Schiff base (Fig. 2). However, it was not the terminal proton acceptor in the M state as, according to FTIR spectroscopy, the C = O stretching frequency for the protonated carboxylate was not observed in the M state^1^. The ground-state structure shows that Glu107 is linked to His80 via a water molecule to form an H-bond network (Fig. 1)^6^.

The potential energy profile for the proton transfer indicates that the proton released from the Schiff base is transferred to His80 via Glu107 (Fig. 2d). Note that when analyzed using the MD-generated structure, the corresponding proton transfer was more energetically uphill due to the over-stabilized Glu107 protonation (Supplementary Fig. 4).

**Fig. 4:**
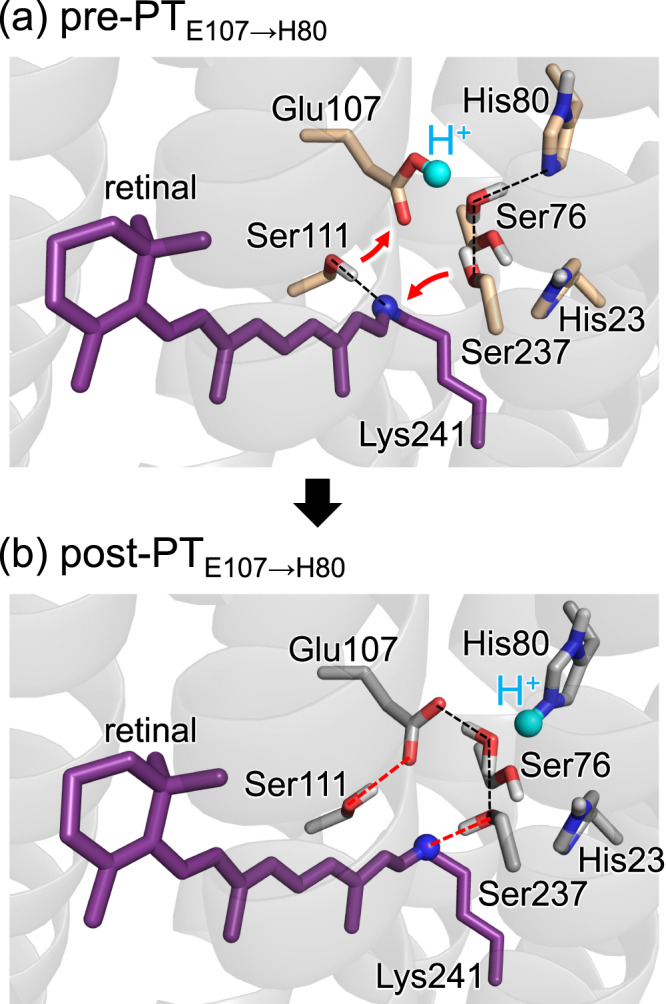
Reorientations of serine residues owing to proton transfer from Glu107 to His80 in QM/MM calculations. **a** Pre-PT_E107→H80_ conformation. **b** Post-PT_E107→H80_ conformation. Dotted lines indicate H-bonds. Red curved arrows indicate the reorientations of serine residues. Red dotted lines indicate H-bond formations induced by proton transfer from Glu107 to His80.

Remarkably, Ser237, which exhibits no conformational change during proton transfer from the Schiff base to Glu107 (Fig. 3a), reorients toward the Schiff base during the proton transfer from Glu107 to His80 (Fig. 4). Overall, the three serine residues, Ser76, Ser111, and Ser237, reorient as the proton moves from the Schiff base toward His80.

It seems possible that the proton at the His80 moiety can be further transferred to His23, as the two histidine residues form an H-bond chain via a water molecule (Fig. 1)^6^. The photocycle was significantly slowed down in the H23F mutant protein^1^. Note that the M state can also form in the H80F and H23F mutants of heliorhodopsin 48C12^6^ and even in the H23F/H82F double mutant of heliorhodopsin from *Thermoplasmatales* archaeon SG8-52-1^2^. It seems possible that another site can act as an alternative terminal proton acceptor in these mutant proteins. Thus, the His80/His23 moiety is a plausible candidate as a terminal proton acceptor in the M state, as proposed by Pushkarev et al.^1^.

### Conformational changes on the extracellular side upon His80/His23 protonation

To investigate the stability of protonation at the terminal proton acceptor, His80/His23, MD simulations were conducted for the ground-state (charge-neutral His80 and His23 with the protonated all-*trans* retinal Schiff base) and M-state (H^+^ at either His80 or His23 with the deprotonated 13-*cis* retinal Schiff base) conformations.

**Fig. 5:**
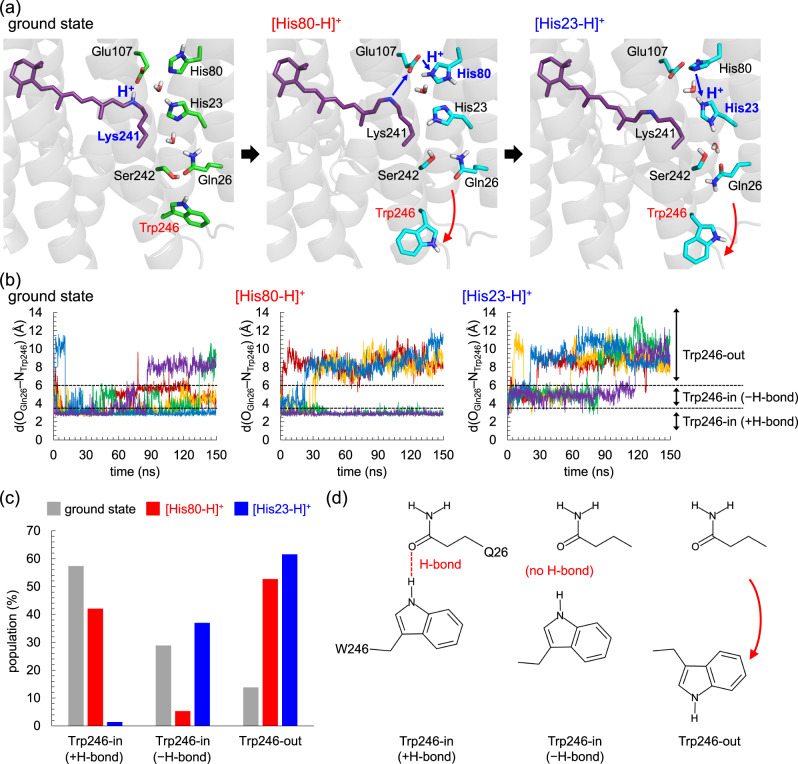
Trp246 reorientation in response to His80/His23 protonation. **a** Transitions of the heliorhodopsin structures from the ground state (left panel) via M state with doubly protonated His80 ([His80-H]^+^, middle panel) to M state with doubly protonated His23 ([His23-H]^+^, right panel). Structures shown in the panels were obtained after 150-ns MD runs. **b** Distances between the side-chain O atom of Gln26 and the side-chain N atom of Trp246 (d(O_Gln26_–N_Trp246_) in Å) during five independent 150 ns MD runs for the ground, [His80-H]^+^, and [His23-H]^+^ states. The Trp246 conformations are classified into the Trp246-in (+H-bond), Trp246-in (−H-bond), and Trp246-out conformations by d(O_Gln26_–N_Trp246_). That is, the Trp246-in (+H-bond) conformation for d(O_Gln26_–N_Trp246_) < 3.5; the Trp246-in (−H-bond) conformation for 3.5 ≤ d(O_Gln26_–N_Trp246_) ≤ 6.0; the Trp246-out conformation for d(O_Gln26_–N_Trp246_) > 6.0. **c** Populations of the Trp246-in (+H-bond), Trp246-in (−H-bond), and Trp246-out conformations observed during the five independent 150 ns MD runs for the ground, [His80-H]^+^, and [His23-H]^+^ states. **d** Trp246-in (+H-bond), Trp246-in (−H-bond), and Trp246-out conformations.

In the MD simulations, Trp246 exhibits two distinct conformations: it orients toward the protein interior (Trp246-in conformation) or toward the extracellular bulk region (Trp246-out conformation) (Fig. 5a, Supplementary Fig. 5). Intriguingly, the Trp246-out conformation is more pronounced in the M state than in the ground state (Fig. 5b–d).

As Trp246 is 15 Å away from His80 in the ground-state structure (i.e., Trp246-in conformation), its reorientation is not only due to the direct (site-to-site) electrostatic interaction with increasing net charge at His80, but also due to the electrostatic interaction that propagates along the transmembrane H-bond network [His80…His23…Gln26…Trp246]. The Trp246 reorientation is more pronounced upon the His23 protonation (Fig. 5c), as the reorientation of the –C=O group of Gln26 toward His23 also occurs and facilitates the Trp246 reorientation (Supplementary Fig. 6). Thus, the –NH_2_ group of Gln26 orients toward the –NH group of Trp246, leading to significant movement of Trp246 upon the His23 protonation (Fig. 5).

Remarkably, the Trp246-out conformation is consistent with the conformation in the structure crystallized at pH = 4.3 (low-pH crystal structure, Supplementary Fig. 7), whereas the initial Trp246-in conformation is consistent with the conformation in the structure crystallized at pH = 8.8 (high-pH crystal structure, Supplementary Fig. 7)^6^. This result suggests that the Trp246-out conformation in the low-pH crystal structure is attributable to the protonation of the His80/His23 moiety.

**Fig. 6:**
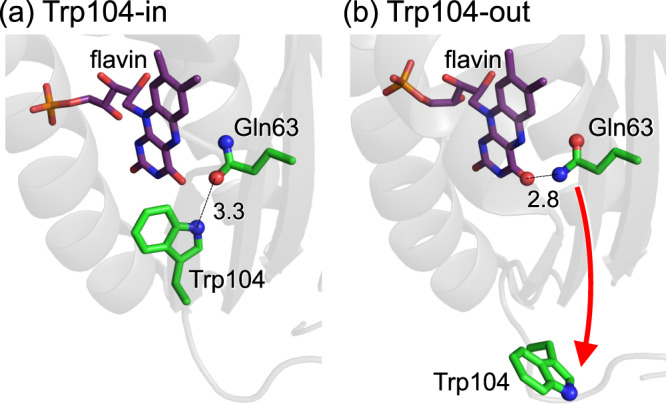
Two Trp104 conformations in AppA BLUF domain. **a** Trp104 orienting toward the flavin moiety (PDB ID 1YRX^17^). **b** Trp104 orienting toward the bulk region (PDB ID 2IYG^18^). Dotted lines indicate H-bonds. Values indicate distances (in Å). The red curved arrow indicates the Trp-in to Trp-out conformational change.

The existence of the Trp246-in and Trp246-out conformations in heliorhodopsin is similar to that of the Trp104-in and Trp104-out conformations in blue light using flavin (BLUF) domain (Fig. 6)^17–20^. Similar to Trp246-in, which forms an H-bond network via Gln26 with the protonation site of His23 in heliorhodopsin, Trp104-in forms an H-bond network via Gln63 with the protonation site of flavin. The light-sensing activity of the BLUF domain is suppressed upon the Q63L^21^ or W104A^21,22^ mutations, which suggests that the H-bond network is crucial for this function. In addition, heliorhodopsin 48C12 has been proposed to function as a photosensor owing to its long photocycle time (>1 s)^1^. The reorientation of tryptophan residues via glutamine may provide a mechanism for the signaling activity commonly used in the two photo-signaling proteins (Figs. 5, 6).

### Structural fluctuation on the intracellular side upon His80/His23 protonation

**Fig. 7:**
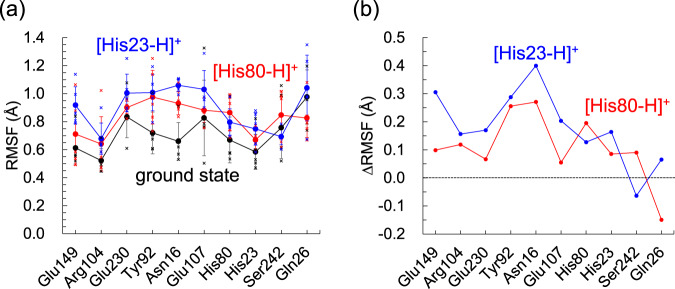
His80/His23 protonation and the fluctuation of the H-bond network. **a** Root mean square fluctuation (RMSF) of the side-chains along the H-bond network of the Schiff base in the ground state (black line) and the M state with doubly protonated His80 (red line) and doubly protonated His23 (blue line) conformations in the range of 0–150 ns. Vertical bars represent standard deviations of five independent MD runs. Crosses indicate RMSF values in each MD run for the ground state (black), the M state with doubly protonated His80 (red), and the M state with doubly protonated His23 (blue) conformations. **b** RMSF with respect to the ground state (ΔRMSF). His23 and His80 are charge-neutral in the ground state.

Protonation at the His80/His23 moiety induces not only conformational change at the extracellular side but also structural fluctuation at the intracellular side. Particularly, histidine protonation induces structural fluctuations in Asn16 (Fig. 7, Supplementary Table 1). The fluctuation of Asn16 is specifically facilitated by histidine protonation because it directly forms an H-bond with His80, forming a salt-bridge with Glu107 during MD simulations in the M state. Indeed, the H-bond between Asn16 and His80, which is stable in the ground-state conformation, is unstable in the M-state conformation due to the large displacement of the His80 side-chain (Supplementary Fig. 8).

**Fig. 8:**
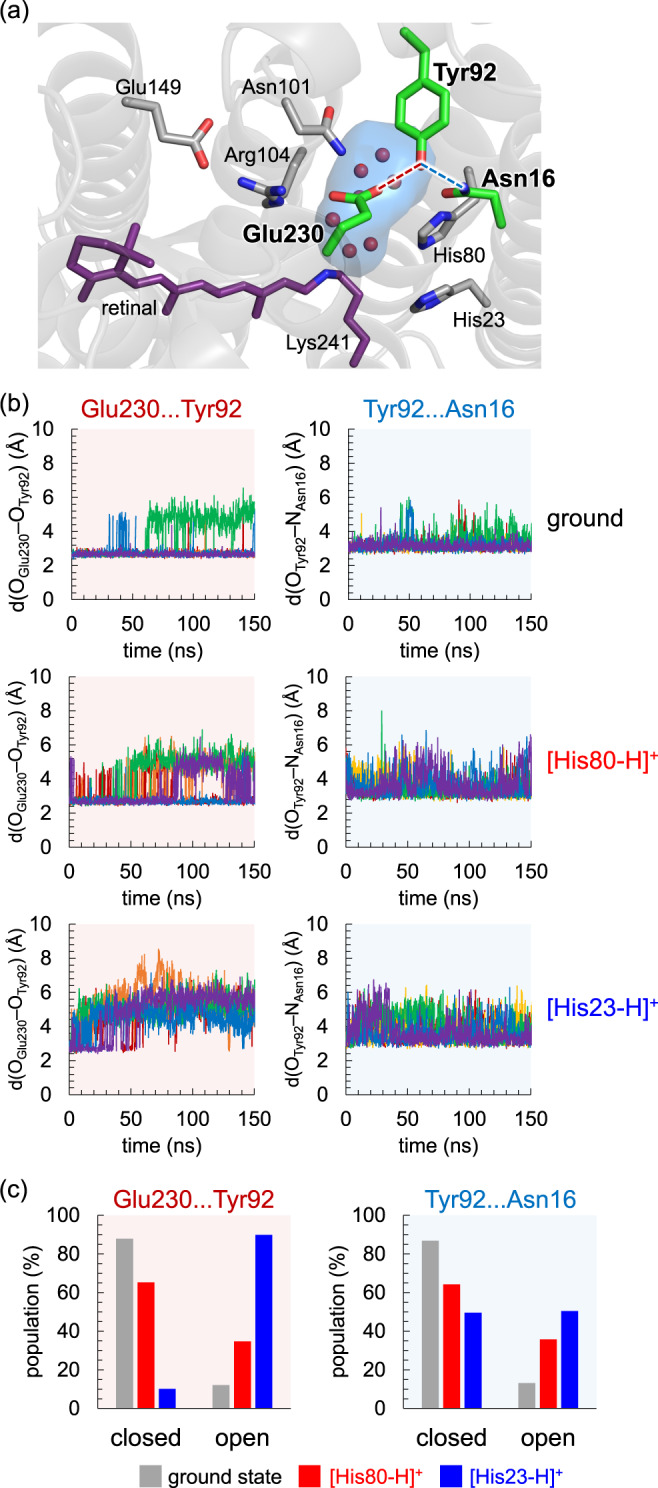
Structural changes in the intracellular region in response to His80/His23 protonation. **a** H-bond network of [Glu230-COO^−^…HO-Tyr92…HN-Asn16]. **b** Distances between the side-chain O atom of Glu230 and the side-chain O atom of Tyr92 (d(O_Glu230_–O_Tyr92_) in Å, left panel) and distances between the side-chain O atom of Tyr92 and the side-chain N atom of Asn16 (d(O_Tyr92_–N_Asn16_) in Å, right panel) during the five independent 150-ns MD runs for the ground state, M state with doubly protonated His80 ([His80-H]^+^), and M state with doubly protonated His23 ([His23-H]^+^). **c** Populations of the “closed” and “open” conformations for [Glu230…Tyr92] pair (left panel) and [Tyr92…Asn16] pair (right panel) in the five independent 150-ns MD runs for the ground, [His80-H]^+^, and [His23-H]^+^ states. The “closed” conformation stands for d(O_Glu230_–O_Tyr92_) < 3.5 and d(O_Tyr92_–N_Asn16_) < 3.5. The “open” conformation stands for d(O_Glu230_–O_Tyr92_) ≥ 3.5 and d(O_Tyr92_–N_Asn16_) ≥ 3.5.

In bacteriorhodopsin, Arg82, which corresponds to Arg104 in heliorhodopsin, facilitates the release of the proton, reorienting the side-chain during the photocycle^23,24^. In contrast, the two NH_2_ sites of Arg104 are fixed by salt-bridges with Glu149 and Glu230 (Fig. 1), rendering the occurrence of the corresponding movement unlikely. The fluctuation of the H-bond network (i.e., Glu149, Arg104, Glu230, Tyr92, and Asn16) may compensate for the energy not consumed by Arg104 in heliorhodopsin. Overall, the pronounced structural fluctuation of the H-bond network adjacent to Arg104 in the M state of heliorhodopsin resembles the movement of Arg82 in bacteriorhodopsin, which may play a role in gating, e.g., possibly conducting ions or molecules, during the photocycle, as observed for Arg82 in bacteriorhodopsin^23,24^. Indeed, the binding of an acetate ion to the Glu107 and Glu230 moieties was observed in the intracellular region of the low-pH crystal structure (Supplementary Fig. 7)^6^. Binding of nitric acid ions was also observed in the E107A and E107Q mutant proteins^25^. The [Glu230-COO^−^…HO-Tyr92…HN-Asn16] H-bond network is substantially stable in the ground state conformation, whereas it is unstable in the M-state conformation (Fig. 8a, b). Remarkably, the pronounced fluctuation of the H-bond network in the M state makes the two H-bonds, [Glu230…Tyr92] and [Tyr92…Asn16], weak: the movement of these sidechains resembles “gates”, opening the protein intracellular surface in the M-state conformation (Fig. 8c). As either His80 or His23 is probably doubly protonated in the low-pH crystal structure, anion binding at the moiety implies that the [Glu230…Tyr92] and [Tyr92…Asn16] pairs play a role in gating the uptake of ions or molecules in response to light-induced proton transfer.

## Discussion

**Fig. 9:**
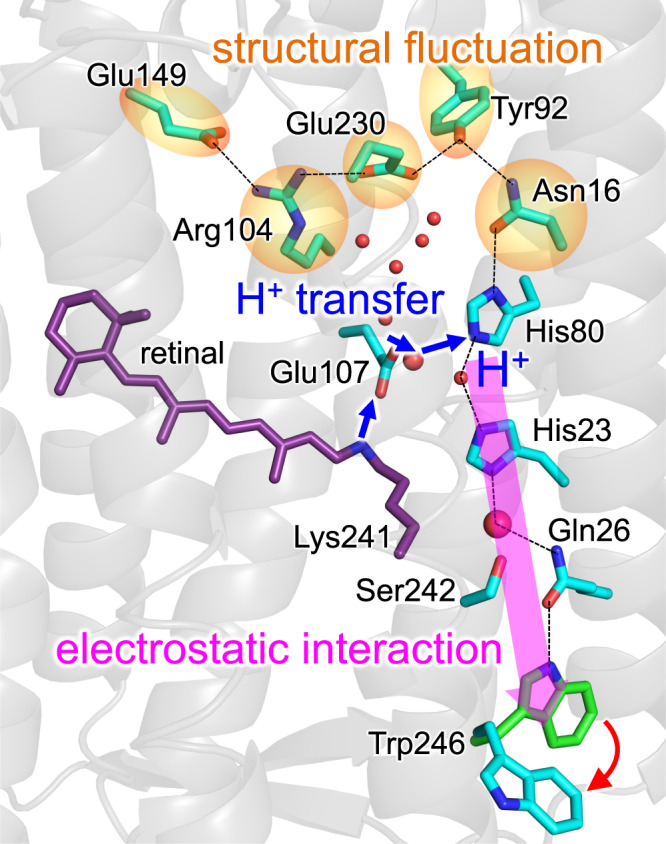
Mechanism of the proton-mediated structural changes in heliorhodopsin. Blue arrows indicate proton transfer. The pink thick arrow indicates the electrostatic interaction along the H-bond network. The red curved arrow indicates the Trp-in to Trp-out conformational change.

The present result indicates that Glu107 and His80 and some water molecules in the cluster of water molecules mediate proton transfer (Fig. 9). The proton-conducting water molecules in the proton transfer pathway resemble the water molecules in BR, in which a proton might be delocalized over a cluster of water molecules near Tyr57, Arg82, Tyr83, Glu194, and Glu204^11,12^. Indeed, Tyr57, Arg82, Tyr83, and Glu194 in BR are conserved as His80, Arg104, Trp105, and Glu149 in heliorhodopsin (Supplementary Fig. 9).

Based on the results presented here, the following mechanism can be deduced for the photoinduced reaction in heliorhodopsin: photoinduced proton transfer proceeds from the Schiff base to Glu107, reorienting Ser76 from Glu107 toward His23 (Fig. 3). The two Ser76 conformations are consistent with those identified in the ground-state crystal structure (Fig. 3)^6^. The proton is easily transferred from Glu107 toward His80 even in the ground-state structure (Fig. 2d), which is in line with FTIR studies that suggested that Glu107 is not the terminal proton acceptor in the M state^1^.

In the M state, His80 protonation leads to a conformational change on the extracellular surface, i.e., the Trp246-in to Trp246-out conformational change (Fig. 5). As Trp246 is 15 Å away from His80 in the ground-state structure (i.e., Trp246-in conformation)^6^, Trp246 reorientation cannot be explained by the direct electrostatic interaction with increasing net charge at His80. The Trp246-in to Trp246-out conformational change is pronounced by the His23 protonation. The protonation of His23 reorients the Gln26 side-chain (Supplementary Fig. 6), the –NH_2_ group of which donated an H-bond to His23 via a water molecule, as the –C = O group accepted an H-bond from the –NH group of Trp246 in the ground-state structure (Fig. 5a).

**Fig. 10:**
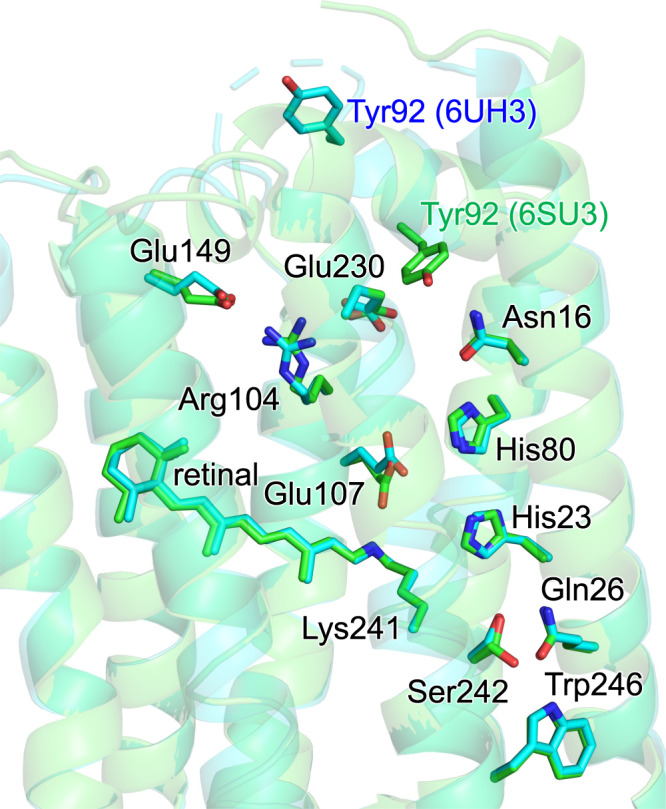
Comparison of two crystal structures of heliorhodopsin 48C12. Root-mean-square deviation: 1.96 Å. Green sticks: PDB ID 6SU3^6^. Cyan sticks: PDB ID 6UH3^7^.

His80 protonation also induces structural fluctuations along the H-bond network His80...Asn16...Tyr92...Glu230...Arg104...Glu149 on the intracellular side (Fig. 7). Arg82 reorients and facilitates proton transfer in bacteriorhodopsin^23^. It seems possible that the corresponding movement is pronounced at the entire H-bond network adjacent to Arg104 in heliorhodopsin, as implied by the alternative side-chain conformation of Tyr92 (Fig. 10)^7^. The loss of the H-bonds [Glu230…Tyr92] and [Tyr92…Asn16] upon the M-state formation opens the intracellular surface, connecting between the cavity filled with a cluster of water molecules and the bulk water region (Fig. 8). Genomic studies suggested that signal-transducing domains are likely adjacent to the intracellular side of heliorhodopsin^5^. It was also reported that heliorhodopsin binds at a glutamine synthetase and upregulates the synthetic activity in the presence of light^26^. The proton-mediated structural fluctuation at the intracellular side may be a microscopic origin of the proposed role of heliorhodopsin as a photosensor or regulator.

## Methods

### Coordinates and atomic partial charges

To the best of our knowledge, heliorhodopsin 48C12 crystal structures are reported at 1.5 Å resolution with pH 8.8 (PDB code 6SU3^6^), 1.5 Å resolution with pH 4.3 (PDB code 6SU4^6^), and 2.7 Å resolution (PDB code 6UH3^7^). Considering the atomic resolution and the pH used for crystallization, the X-ray structure of heliorhodopsin 48C12 monomer unit “A” (PDB code 6SU3^6^) was used as the initial structure for the protonation pattern calculation, MD simulation, and QM/MM calculation. All crystal water molecules were included explicitly in calculations if not otherwise specified. During the optimization of hydrogen atom positions with CHARMM^27^, the positions of all heavy atoms were fixed, and all titratable groups (e.g., acidic and basic groups) were ionized. The Schiff base was considered protonated. Atomic partial charges and force field parameters of the amino acids were obtained from the CHARMM22^28^ parameter set. Atomic charges and force field parameters of the protonated all-*trans* and deprotonated 13-*cis* retinal Schiff base (retinol and retinal parameters, A. Loccisano, A.M.W., J. Evanseck, and A.D. MacKerell, Jr., 2005) were obtained from CHARMM-GUI^29^.

### Protonation pattern

The computation was based on the electrostatic continuum model, solving the linear Poisson-Boltzmann equation with the MEAD program^30^. The difference in the p*K*_a_ value of the protein relative to the reference system, which corresponds to the difference in electrostatic energy between the protonated and deprotonated states in a reference model system, was calculated and then added to the known reference p*K*_a_ value. The experimentally measured p*K*_a_ values employed as references were 12.0 for Arg, 4.0 for Asp, 9.5 for Cys, 4.4 for Glu, 10.4 for Lys, 9.6 for Tyr^31^, and 7.0 and 6.6 for the N_ε_ and N_δ_ atoms of His, respectively^32–34^. All other titratable sites were fully equilibrated to the protonation state of the target site during titration. The dielectric constants were set to 4 for the protein interior and 80 for water. All water molecules were considered implicitly. All computations were performed at 300 K, pH 7.0, and with an ionic strength of 100 mM. The linear Poisson-Boltzmann equation was solved using a three-step grid-focusing procedure at resolutions of 2.5, 1.0, and 0.3 Å, yielding the intrinsic p*K*_a_ values (p*K*_a int_) for all titratable sites in the protein. p*K*_a int_ is the p*K*_a_ value obtained in the presence of all titratable sites being in the charge neutral states (i.e., protonated acidic and deprotonated basic residues). The protonation probability <*x*_*i*_> of the *i*-th titratable site in the protein was calculated using Eq. 1

1 $$\langle {x}_{i}\rangle =\frac{\mathop{\sum}\limits_{q}{x}_{i}\exp \left[-{\beta }\mathop{\sum}\limits_{\mu }\left({{x}}_{\mu }\Delta {{G}}_{{{{{{\rm{int}}}}}},\mu }+\frac{1}{2}\mathop{\sum}\limits_{{\nu }\ne \mu }{{q}}_{\mu }{{q}}_{{\nu }}{{W}}_{\mu {\nu }}\right)\right]}{\mathop{\sum}\limits_{{q}}\exp \left[{-}{\beta }\mathop{\sum}\limits_{\mu }\left({{x}}_{\mu }\Delta {{G}}_{{{{{{\rm{int}}}}}},\mu }+\frac{1}{2}\mathop{\sum}\limits_{{\nu }\ne \mu }{{q}}_{\mu }{{q}}_{{\nu }}{{W}}_{\mu {\nu }}\right)\right]}$$

where *β* is 1/(*k*_B_*T*), *k*_B_ is the Boltzmann constant, *T* is the temperature, *q*_*i*_ is the net charge of the *i*-th titratable site, *ΔG*_int,*i*_ is the protonation energy for the *i*-th titratable site (corresponding to p*K*_a int,*i*_), and *W*_*μν*_ is the electrostatic interaction between the *μ*-th and *ν*-th titratable sites. *x*_*i*_ is 1 and 0 for the protonated and deprotonated *i*-th titratable site, respectively. To solve Eq. 1, the ensemble of the protonation pattern was sampled by the Monte Carlo method with the Karlsberg program^35^. Note that the protonation pattern was consistent with that calculated using the PROPKA 3^36–39^ program (Supplementary Table 2).

### MD simulations

The heliorhodopsin assembly was embedded in a lipid bilayer consisting of 281 1-palmitoyl-2-oleyl-sn-glycero-3-phosphocholine (POPC) molecules using CHARMM-GUI^29^, and soaked in 33,362–33,367 TIP3P water models. 51 sodium and 57 chloride ions were added to neutralize the system with an ionic strength of 100 mM using the VMD plugins^40^ (Supplementary Table 3). After structural optimization with position restraints on heavy atoms of the heliorhodopsin assembly, the system was heated from 0.1 to 300 K over 5.5 ps with time step of 0.1 fs, equilibrated at 300 K for 1 ns with time step of 0.5 fs, and annealed from 300 to 0 K over 5.5 ps with time step of 0.1 fs. The positional restraints on heavy atoms of side-chains were released, and the same procedure was repeated. Positional restraints on any atoms were released, and the system was heated from 0.1 K to 300 K over 5.5 ps with time step of 0.1 fs and equilibrated at 300 K for 1 ns with time step of 0.5 fs. The system was equilibrated at 300 K for 150 ns with time step of 1.5 fs. The coordinates were obtained every 0.15 ns. Five independent MD runs, instead of enhanced sampling method, are conducted for each protonation state to properly analyze the side-chain conformations in the H-bond network of the Schiff base. All MD simulations were conducted with the CHARMM22^28^ force field parameter set using the MD engine NAMD version 2.11^41^. For MD simulations with time step of 1.5 fs, the SHAKE algorithm for hydrogen constraints was employed^41^. For temperature and pressure control, the Langevin thermostat and piston were used^42,43^.

### QM/MM calculations

We employed the electrostatic embedding QM/MM implemented in the QSite^44^ program (Schrödinger Inc.). Electrostatic and steric effects of the protein environment were explicitly considered. The intra-molecule interface between the QM and MM regions was treated as an H cap: the terminal atom in the QM region singly-bonded with a MM atom was capped with an H atom. To investigate the structural change of the local H-bond network, the geometry of all atoms in the QM region, in which QM interactions were accurately described, was fully optimized. In contrast, the MM force field cannot describe QM interactions. To avoid irrelevant and artificial conformational changes of the protein backbone due to an inadequacy of the MM force field, the heavy atoms were fixed and only hydrogen atoms were optimized in the MM region.

The geometry was optimized using a QM/MM approach. In the present study, the restricted density functional theory (DFT) method was employed with the B3LYP functional and LACVP* basis sets using the QSite^44^ program.

The QM region was defined as the retinal, Schiff base (including the Lys241 side-chain), the side-chains of Asn16, His23, Ser76, His80, Arg104, Glu107, Tyr108, Ser111, Glu230, and Ser237, and the water molecules at the Schiff base moiety (H_2_O-916, 919, 927, 939, 944, 969, 1005, 1007^6^) (Supplementary Fig. 11). All atomic coordinates were fully relaxed in the QM region, and the protonation pattern of titratable residues was implemented in the atomic partial charges of the corresponding MM region. In the MM region, the positions of H atoms were optimized using the OPLS2005 force field^45^, while the positions of the heavy atoms were fixed. See Supplementary Data 1 for the atomic coordinates of the QM/MM-optimized structures.

The QM/MM approach was also used to analyze the potential energy profiles for the proton transfer (see below). The shape of the potential-energy profile for proton transfer did not significantly depend on the theory used in the QM region (e.g., the density functional theory (DFT) and the complete active space self-consistent field second-order perturbation theory (CASPT2)) or the functional/basis sets (e.g., B3LYP and CAM-B3LYP for functional/6–31 G* and 6–31 G** for basis sets) in microbial rhodopsins (Supplementary Fig. 10). To obtain the potential energy profiles for the proton transfer, the QM/MM-optimized geometry was used as the initial geometry. The H atom under investigation was moved from the H-bond donor atom (D) toward the acceptor atom (A) by 0.05 Å, after which the geometry was optimized by constraining the H–A distance, and the energy was calculated. The H…A distance was decreased systematically, using the distance constraint option in QSite. These procedures were repeated until the H atom reached the A atom. All atomic coordinates were fully relaxed in the QM region, whereas only the H atom positions were optimized in the MM region.

The absorption energy of microbial rhodopsins is highly correlated with the lowest excitation energy of the retinal Schiff base calculated using time-dependent (TD) DFT (*E*_TD-DFT_)^46,47^. To calculate absorption energies and corresponding wavelengths, the lowest excitation energies were calculated. The absorption energy (*E*_abs_ in eV) was calculated using the following equations, which are obtained for 13 microbial rhodopsins^46^.

2 $${E}_{{{{{{\rm{abs}}}}}}}=1.754\,{E}_{{{{{{\rm{TD}}}}}}-{{{{{\rm{DFT}}}}}}}\,-\,2.073$$

A QM/MM approach utilizing the polarizable continuum model (PCM) method with a dielectric constant of 78 for the bulk region, in which electrostatic and steric effects created by a protein environment were explicitly considered in the presence of bulk water, was employed to calculate the absorption energies. In the PCM method, the polarization points were placed on the spheres with a radius of 2.8 Å from the center of each atom to describe possible water molecules in the cavity. The radii of 2.8–3.0 Å from each atom center and the dielectric constant values of ~80 are likely to be optimal to reproduce the excitation energetics, as evaluated for the polarizable QM/MM/PCM approach^48^. The TD-DFT method with the B3LYP functional and 6-31 G* basis sets was employed using the GAMESS program^49^. The electrostatic contribution of the side-chain in the MM region to the absorption wavelength of the retinal Schiff base was obtained from the shift in the excitation energy upon the removal of the atomic charges of the focusing side-chain.

### Statics and reproducibility

Five independent MD simulations are conducted for ground-state, M-state with doubly protonated His80, and M-state with doubly protonated His23 conformations, respectively. Populations of the Trp246 in/out conformations (Fig. 5c) and the closed/open conformations for [Glu230…Tyr92] and [Tyr92…Asn16] pairs (Fig. 8c) are obtained from the coordinates of five independent MD runs (1000 coordinates from each MD run, 5000 coordinates in total). RMSF values of the side-chains along the H-bond network of the Schiff base are obtained by averaging the RMSF values in the five independent MD runs (Fig. 7). Original RMSF values and standard deviations are also shown in Fig. 7a.

### Reporting summary

Further information on research design is available in the Nature Portfolio Reporting Summary linked to this article.

## Supplementary information

Supplementary Information

Description of Additional Supplementary Files

Supplementary Data 1

Supplementary Data 2

Supplementary Data 3

Reporting Summary

## Data Availability

Source data for Figs. 2c, d, 5b, c, 7 and 8 are provided in Supplementary Data 1. Coordinates for the QM-MM optimized structures of pre-PT_SB→E107_, post-PT_SB→E107_, pre-PT_E107→H80_, and post-PT_E107→H80_ conformations are provided in Supplementary Data 2. NAMD input file and the coordinates at 0 ns and 150 ns in the MD simulations are provided in Supplementary Data 3. All other data are available from the corresponding author upon reasonable request.

## Code availability

Software and algorithms used in this study are available from the following link. CHARMM (https://www.charmm.org), Karlsberg (http://agknapp.chemie.fu-berlin.de/karlsberg_old/), CHARMM-GUI (https://www.charmm-gui.org), VMD (https://www.ks.uiuc.edu/Research/vmd/), NAMD (https://www.ks.uiuc.edu/Research/namd/), QSite (https://www.schrodinger.com/products/qsite), and GAMESS (http://www.msg.chem.iastate.edu/gamess/).
