# Supplementary material for: Proton transfer and conformational changes along the hydrogen bond network in heliorhodopsin
Source: Commun Biol. 2022 Dec 6;5:1336. doi: 10.1038/s42003-022-04311-x (PMC9726877; doi:10.1038/s42003-022-04311-x)
Supplement: Supplementary file 1 — Supplementary Information [file 42003_2022_4311_MOESM1_ESM.pdf]

Supplementary Information

Proton transfer and conformational changes along the hydrogen bond network in heliorhodopsin

Masaki Tsujimura [1]*, Yoshihiro Chiba [2], Keisuke Saito [2,3], and Hiroshi Ishikita [2,3]*

1) Department of Advanced Interdisciplinary Studies, The University of Tokyo, 4-6-1 Komaba, Meguro-ku, Tokyo 153-8904, Japan

2) Department of Applied Chemistry, The University of Tokyo, 7-3-1 Hongo, Bunkyo-ku, Tokyo 113-8654, Japan

3) Research Center for Advanced Science and Technology, The University of Tokyo, 4-6-1 Komaba, Meguro-ku, Tokyo 153-8904, Japan

CORRESPONDING AUTHOR: Tsujimura and Ishikita, Graduate School of Engineering, The University of Tokyo, The University of Tokyo, 4-6-1 Komaba, Meguro-ku, Tokyo 153-8904, Japan, Tel. +81-3-5452-5056, Fax. +81-3-5452-5083, **E-mail:** mtsujimura@protein.rcast.u-tokyo.ac.jp, hiro@appchem.t.u-tokyo.ac.jp

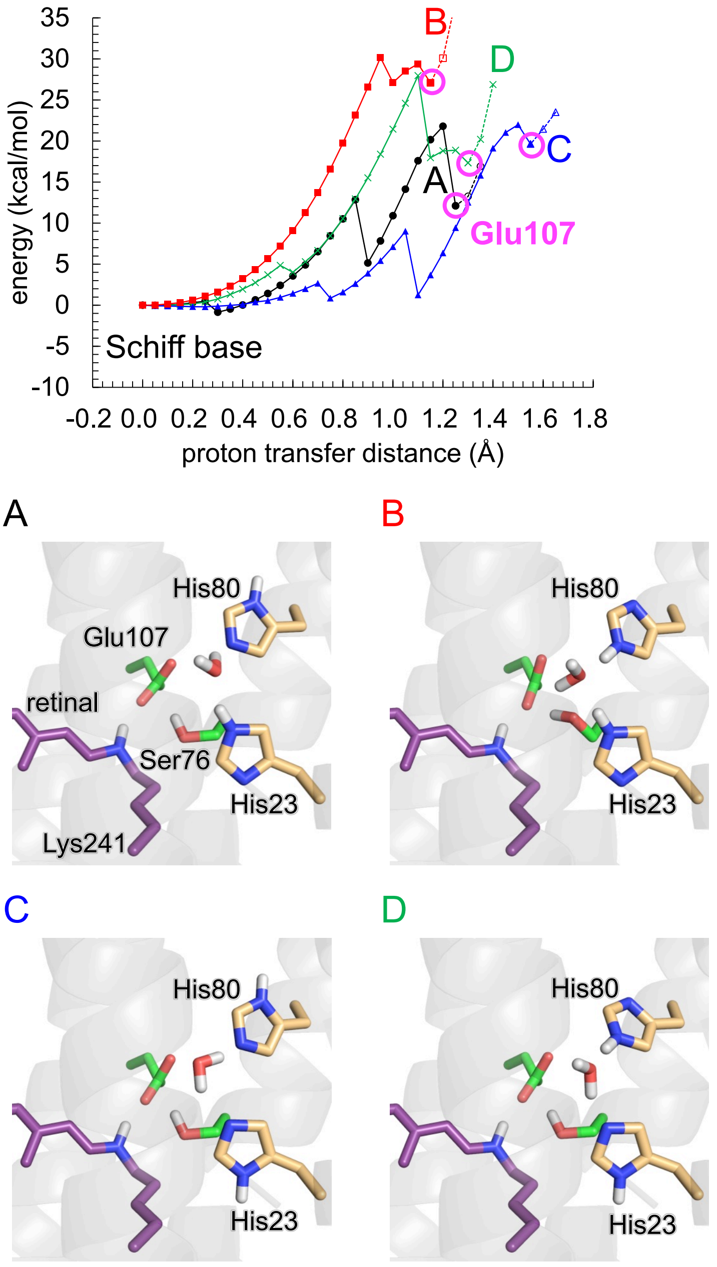

Supplementary Figure 1. Potential energy profile for the proton transfer from the Schiff base to Glu107 in the ground state structure of heliorhodopsin in the presences of (A) [N_δ–His23–N_εH] and [HN_δ–His80–N_ε] (black circle, also shown in Figure 2c), (B) [N_δ–His23–N_εH] and [N_δ–His80–N_εH] (red square), (C) [HN_δ–His23–N_ε] and [HN_δ–His80–N_ε] (blue triangle), and (D) [HN_δ–His23–N_ε] and [N_δ–His80–N_εH] (green cross). The local energy minima with protonated Glu107 are pink circled. Residues at the Schiff base moiety in the QM/MM-optimized structure for each protonation state are shown in the lower panel.

The [N_δ–His23–N_εH] and [HN_δ–His80–N_ε] conformation (A), which has the most stable post-PT structure with protonated Glu107, was used for further analysis.

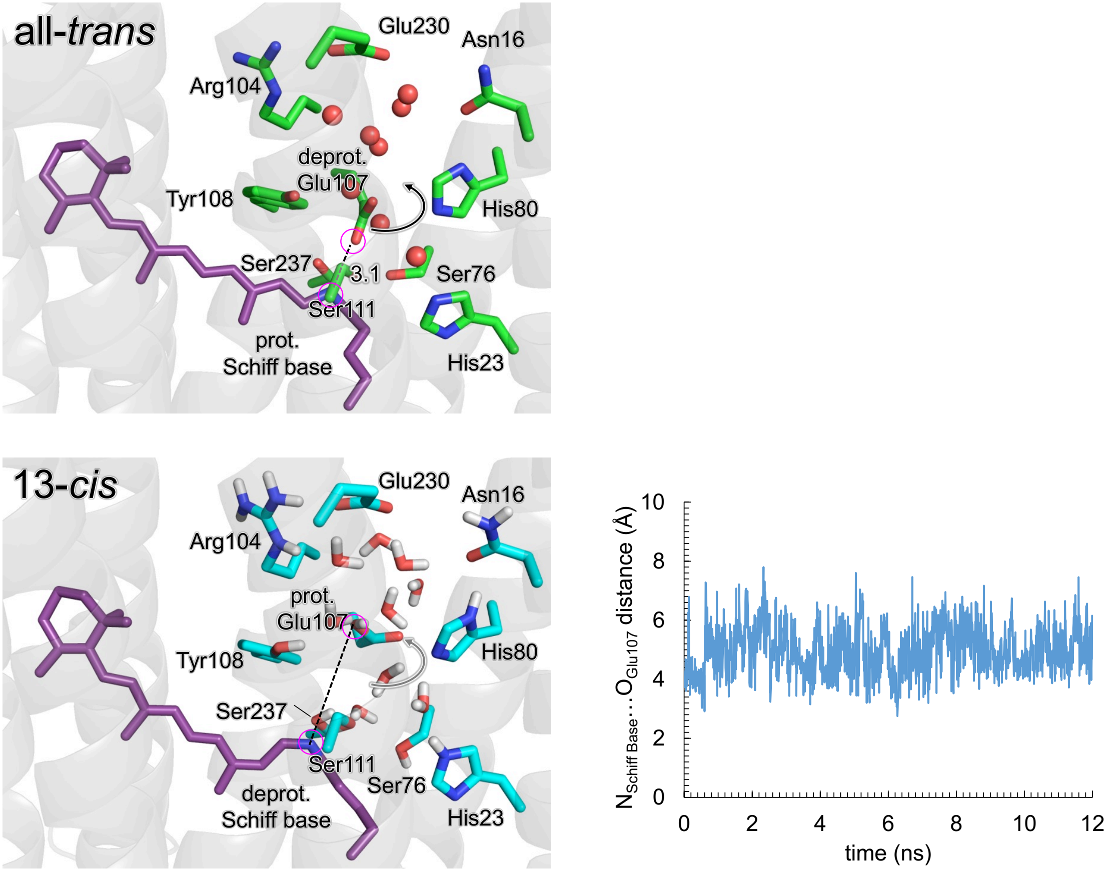

Supplementary Figure 2. All-*trans* (upper, PDB code 6SU3) and 13-*cis* (bottom, MD-generated) retinal conformations. Dotted lines indicate the distance between the Schiff base N atom and the Glu107 O atom. The right panel shows the N…O distance between the Schiff base and Glu107 during the MD simulation.

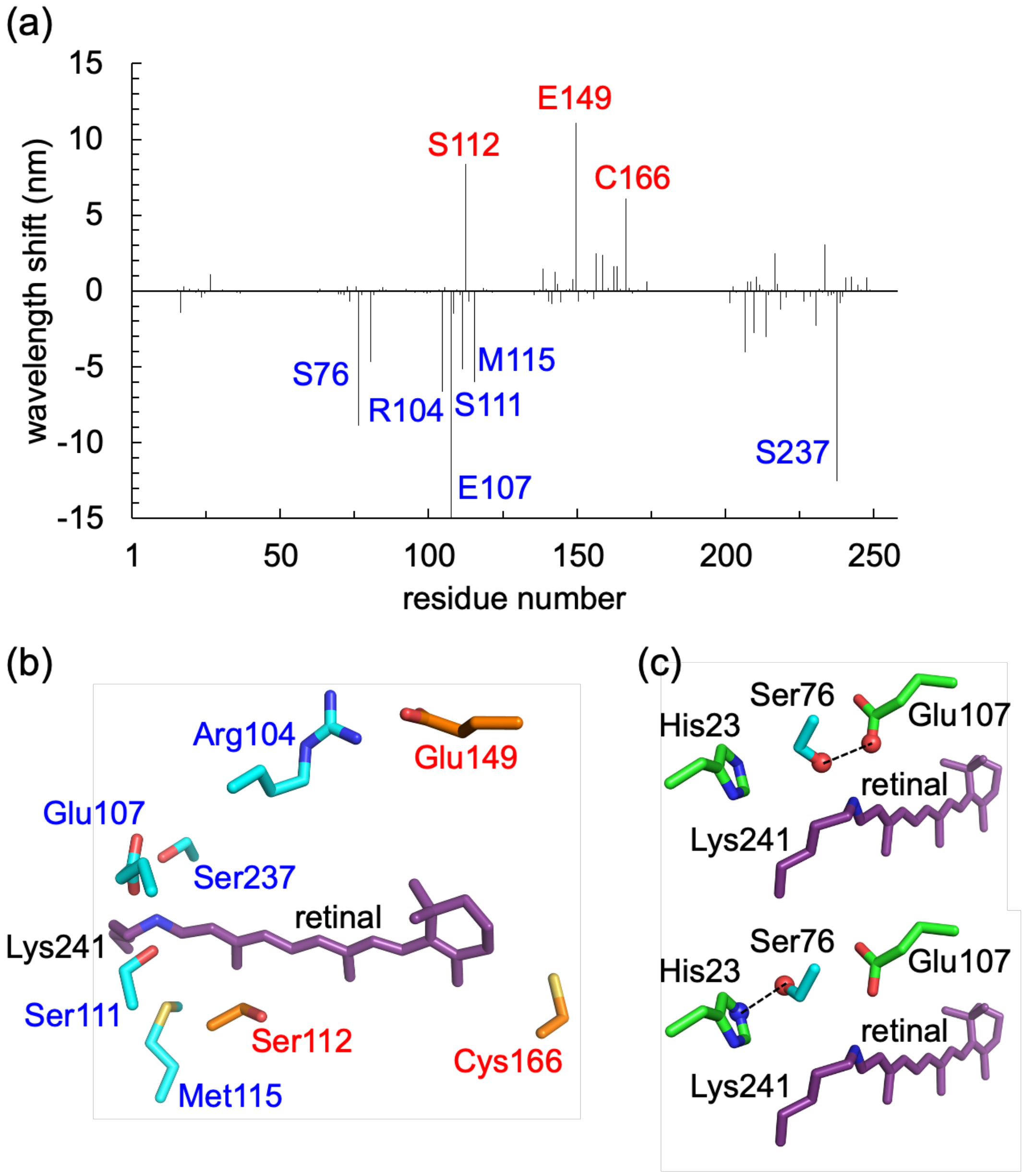

Supplementary Figure 3. Electrostatic contributions of residues to the absorption wavelength. (a) Calculated electrostatic contributions of residues to the absorption wavelength in heliorhodopsin 48C12. Residues which increase and decrease the absorption wavelength by >5 nm are red and blue labeled, respectively. (b) Residues which increase or decrease the absorption wavelength by >5 nm. Residues that increase and decrease the absorption wavelength are red and blue labeled, respectively. (c) Two conformations of Ser76 that orient toward Glu107 (upper panel) or His23 (lower panel) present in the X-ray structure (PDB ID 6SU3 [1]).

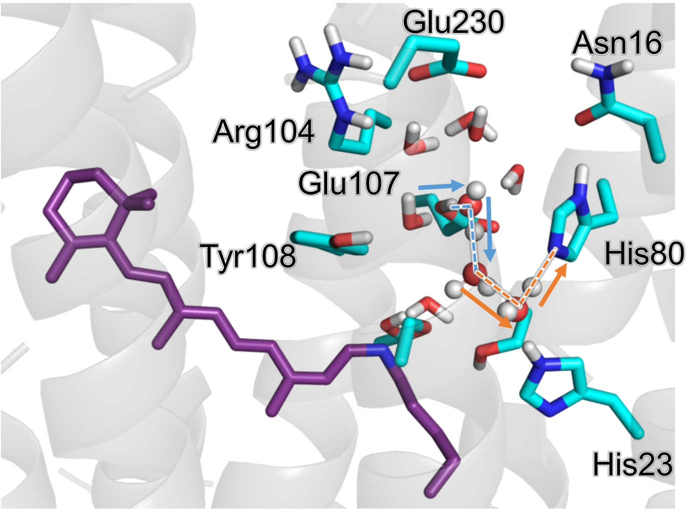

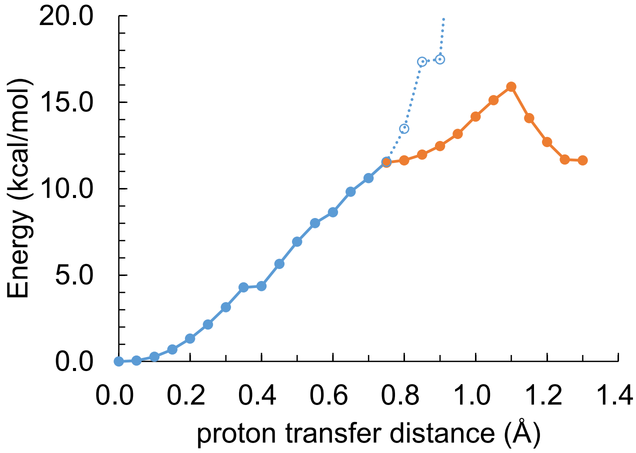

Supplementary Figure 4. Potential energy profile for proton transfer from Glu107 to His80 obtained using the MD-generated structure. Blue arrows indicate proton transfer from Glu107 to the adjacent water molecule. Orange arrows indicate proton transfer from the water molecule to His80.

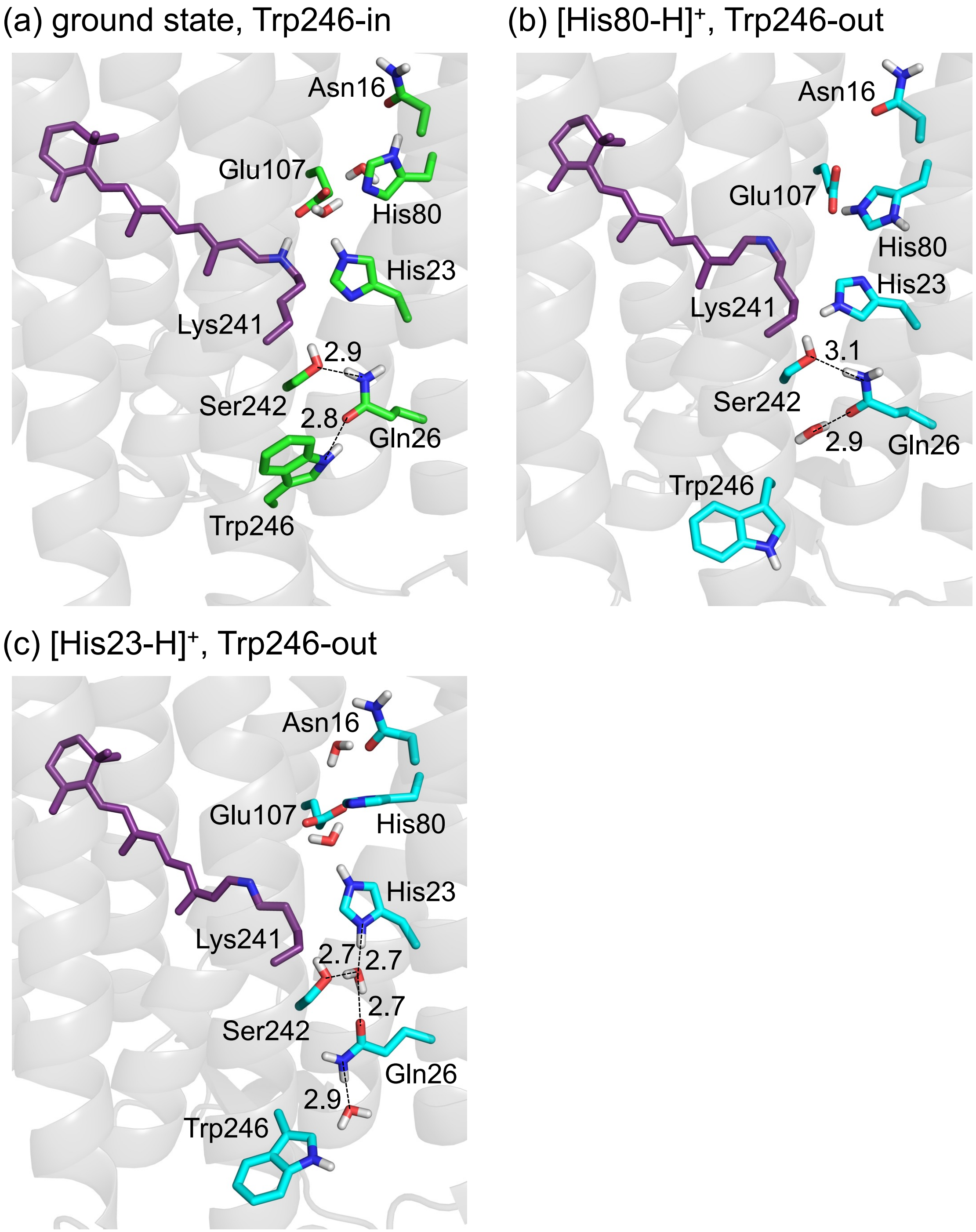

Supplementary Figure 5. QM/MM-optimized structures obtained using MD-generated structures. (a) Ground state in the Trp246-in conformation. (b) M state with doubly protonated His80 in the Trp246-out conformation. (c) M state with doubly protonated His80 in the Trp246-out conformation. The QM region is defined as the side-chains of Asn16, His23, Gln26, His80, Glu107, Ser242, and Trp246 and the water molecules near the H-bond network of Gln26. Dotted lines indicate H-bonds. Values indicate distances (in Å).

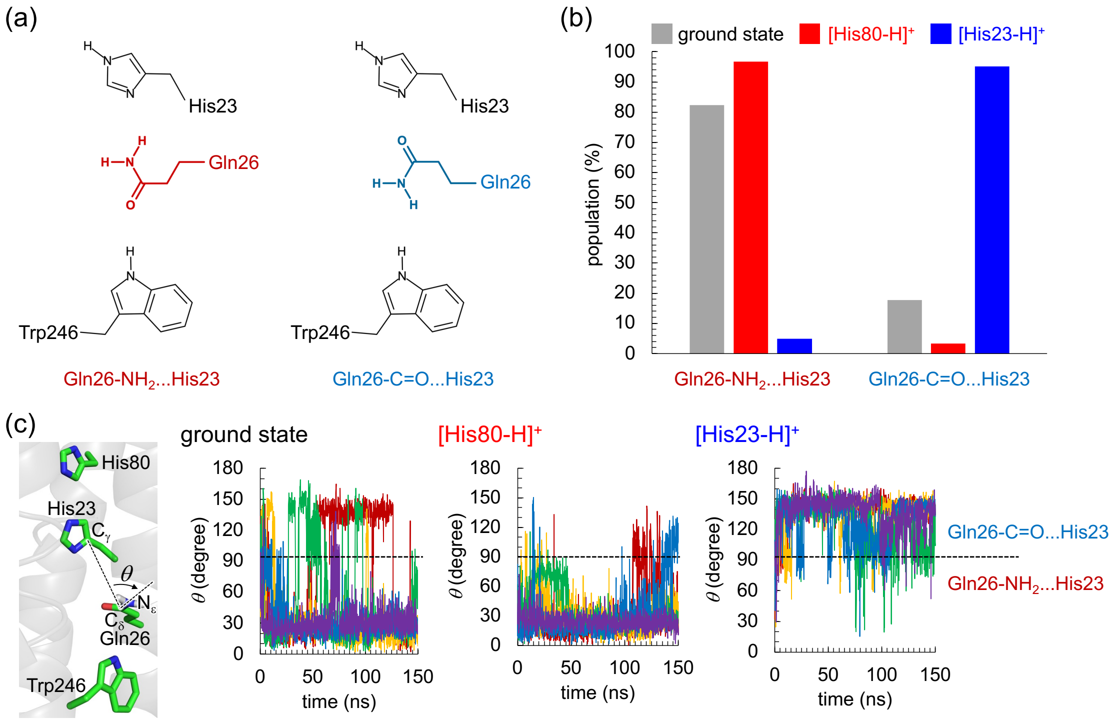

Supplementary Figure 6. Gln26 conformations. (a) Gln26 conformation in which the –NH_2 group orients toward the His23 moiety (Gln26-NH_2...His23 conformation) and the C=O group orients toward the His23 moiety (Gln26-C=O...His23). (b) Populations of the Gln26-NH_2...His23 and the Gln26-C=O...His23 conformations in the five independent 150-ns MD runs for the ground state, the M state with doubly protonated His80 ([His80-H]$^+$), and the M state with doubly protonated His23 ([His23-H]$^+$). (c) $C_{\gamma,His23}$...$C_{\delta,Gln26}$...$N_{\varepsilon,Gln26}$ angle (θ) during the five independent 150 ns MD runs for the ground, [His80-H]$^+$, and [His23-H]$^+$ states. The Gln26 conformation is the Gln26-NH_2...His23 conformation when $\theta < 90°$. If not, the Gln26 conformation is the Gln26-C=O...His23 conformation.

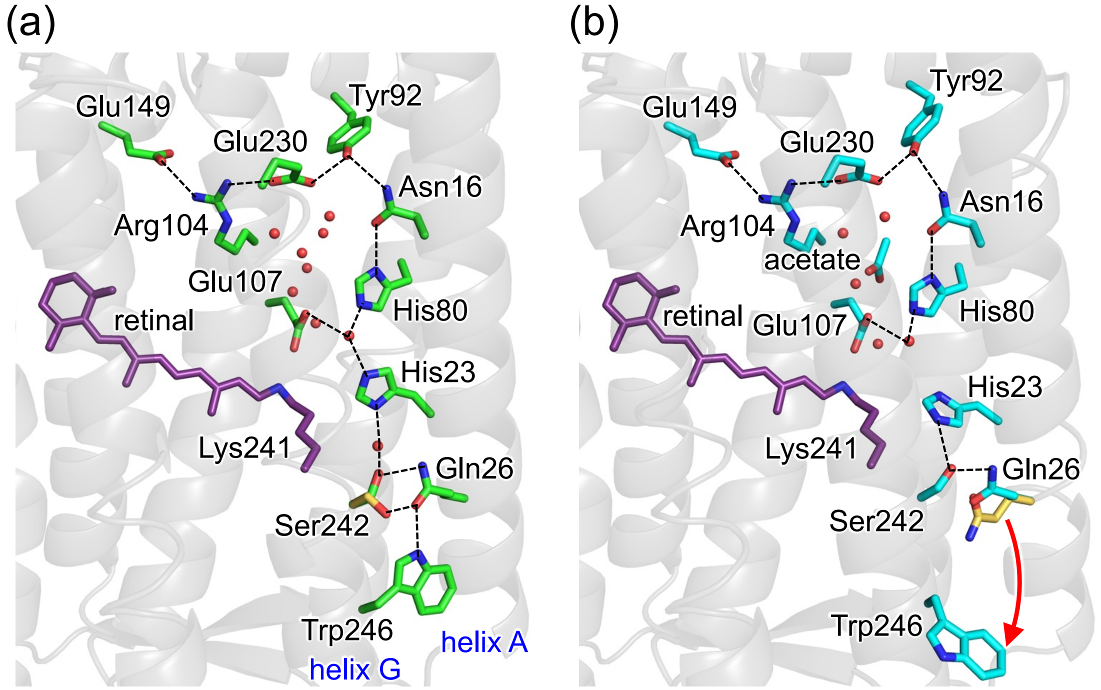

Supplementary Figure 7. Difference in the H-bond network between the low-pH and high-pH structures. (a) High-pH structure (PDB ID 6SU3 [1]). (b) Low-pH structure (PDB ID 6SU4 [1]). Alternative conformations of Gln26 and Ser242 are depicted as green/cyan and yellow sticks. Dotted lines indicate H-bonds. The red curved arrow indicates the Trp-in to Trp-out conformational change.

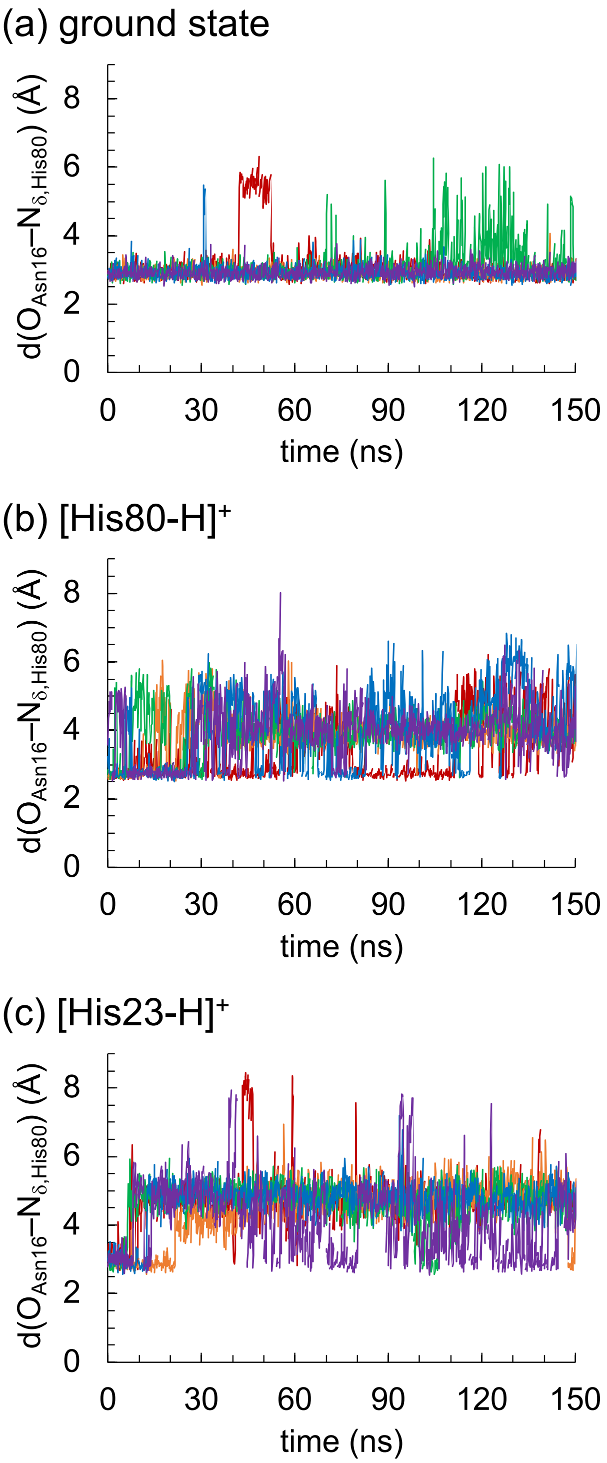

Supplementary Figure 8. Distances between the O atom of the Asn16 side-chain and the $N_δ$ atom of the His80 side-chain (d(O_{Asn16}–$N_{δ,His80}$) in Å) during five independent 150-ns MD runs. (a) Ground state. (b) M state with doubly protonated His80 ([His80-H]$^+$). (c) M state with doubly protonated His23 ([His23-H]$^+$). The H-bond exists between Asn16 and His80 when d(O_{Asn16}–$N_{δ,His80}$) ~ 3.

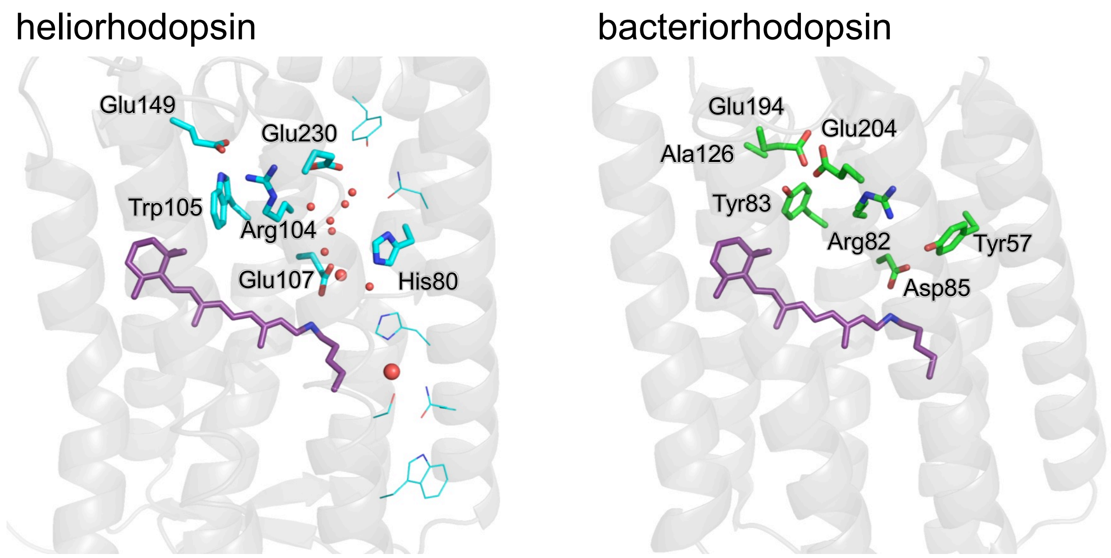

Supplementary Figure 9. Comparison between heliorhodopsin and bacteriorhodopsin. Residues near a cluster of water molecules in heliorhodopsin (PDB ID 6SU3, left panel). Residues in the corresponding region of bacteriorhodopsin (PDB ID 1C3W, right panel).

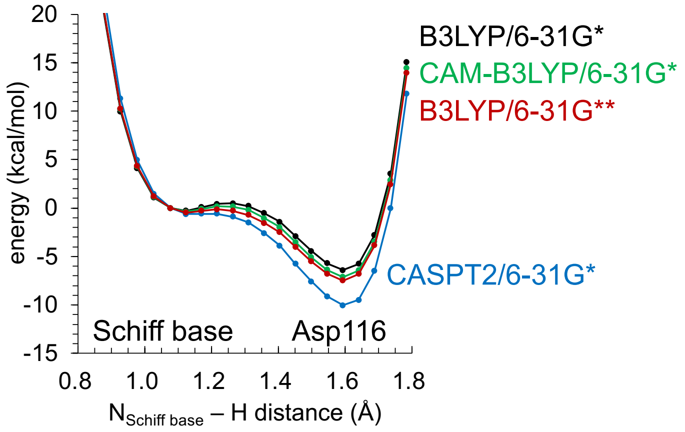

Supplementary Figure 10. Potential-energy profiles for the H-bond between the Schiff base and the counterion (Asp116) in microbial rhodopsin KR2 calculated using the DFT method (with B3LYP/6-31G*, CAM-B3LYP/6-31G*, and B3LYP/6-31G** functional/basis sets) and the CASPT2 method/6-31G* basis set [2,3].

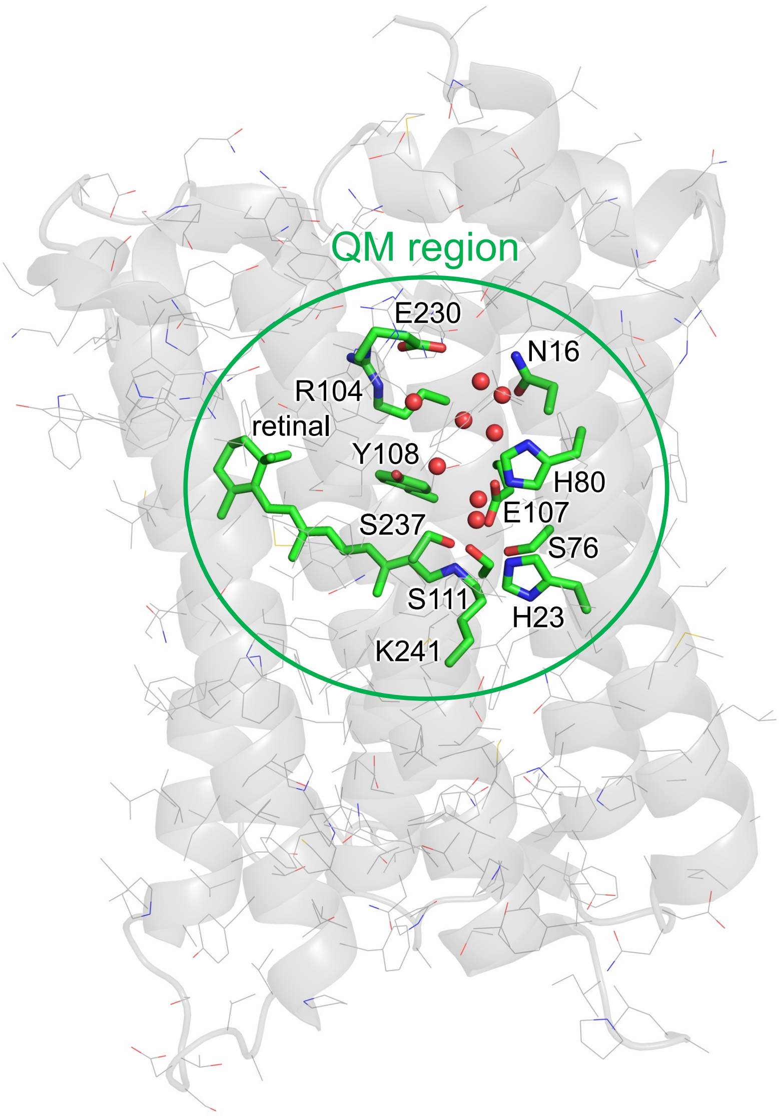

Supplementary Figure 11. QM region investigated for the release of the proton from the Schiff base toward the histidine residues. Residues in the QM region are shown as green sticks. Water oxygen atoms in the QM region are shown as red spheres.

Supplementary Table 1. Average RMSF values in the five independent MD runs of the side-chains along the H-bond network of the Schiff base in ground- and M-state conformations at 0–150 ns. ΔRMSF denotes the RMSF value with respect to the ground-state conformation.

**Groups**	**ground state**	**M state ([His80-H]$^+$)**		**M state ([His23-H]$^+$)**	
			**ΔRMSF**		**ΔRMSF**
Glu149	0.61	0.71	0.10	0.92	0.31
Arg104	0.52	0.64	0.12	0.68	0.16
Glu230	0.83	0.90	0.07	1.00	0.17
Tyr92	0.72	0.97	0.26	1.01	0.29
Asn16	0.66	0.93	0.27	1.06	0.40
Glu107	0.83	0.88	0.05	1.03	0.20
His80	0.67	0.86	0.20	0.80	0.13
His23	0.58	0.67	0.08	0.75	0.16
Ser242	0.76	0.85	0.09	0.69	−0.06
Gln26	0.98	0.83	−0.15	1.04	0.07
Trp246	1.90	1.67	−0.23	2.20	0.30

Supplementary Table 2. Protonation pattern of titratable sites at pH 7 calculated using the MEAD Karlsberg and PROPKA 3 programs.

residue	MEAD/Karlsberg	PROPKA 3
Asp36	deprotonated	deprotonated
Asp127	deprotonated	deprotonated
Asp156	deprotonated	deprotonated
Asp158	deprotonated	deprotonated
Asp188	deprotonated	deprotonated
Glu8	deprotonated	deprotonated
Glu62	deprotonated	deprotonated
Glu107	deprotonated	deprotonated
Glu149	deprotonated	deprotonated
Glu230	deprotonated	deprotonated
Lys150	protonated	protonated
Lys218	protonated	protonated
Arg17	protonated	protonated
Arg104	protonated	protonated
Arg228	protonated	protonated
Arg231	protonated	protonated
His23	neutral (N_δ–His23–N_εH)	neutral
His80	neutral (HN_δ–His80–N_ε)	neutral

Supplementary Table 3. System setup for the MD simulations.

	**ground state**	**M state ([His80-H]$^+$)**	**M state ([His23-H]$^+$)**
**number of atoms**	141931	141946	141934
**number of water molecules**	33362	33367	33363
**number of POPC molecules**	281	281	281
**salt concentration (mM)**	100	100	100

Supplementary References

1 Kovalev, K. *et al.* High-resolution structural insights into the heliorhodopsin family. *Proc. Natl. Acad. Sci. U. S. A.* **117**, 4131-4141 (2020).

2 Tsujimura, M. & Ishikita, H. Identification of intermediate conformations in the photocycle of the light-driven sodium-pumping rhodopsin KR2. *J. Biol. Chem.*, 100459 (2021).

3 Tsujimura, M., Tamura, H., Saito, K. & Ishikita, H. Absorption wavelength along chromophore low-barrier hydrogen bonds. *iScience* **25**, 104247 (2022).
