# Supplementary material for: Proton transfer and conformational changes along the hydrogen bond network in heliorhodopsin
Source: Commun Biol. 2022 Dec 6;5:1336. doi: 10.1038/s42003-022-04311-x (PMC9726877; doi:10.1038/s42003-022-04311-x)
Supplement: Supplementary file 2 — Description of Additional Supplementary Files [file 42003_2022_4311_MOESM2_ESM.pdf]

Description of Additional Supplementary Files

File name: Supplementary Data 1
Description: The source data behind the graphs in the paper.

File name: Supplementary Data 2
Description: Coordinates of the QM/MM-optimized structures shown in Figures 3 and 4.

File name: Supplementary Data 3
Description: NAMD input file and the coordinates at 0 ns and 150 ns in the MD simulations.
